# Metacognition, emotional dysregulation, psychosocial functioning and subjective well-being after 6 months of CBT treatment in pharmacologically stabilized schizophrenic patients

**DOI:** 10.1192/j.eurpsy.2023.804

**Published:** 2023-07-19

**Authors:** F. Raffone, A. Orrico, M. D’Orsi, S. Ferro, M. Russo, V. Martiadis

**Affiliations:** Department of Mental Health, ASL Napoli 1 Centro, Napoli, Italy

## Abstract

**Introduction:**

Psychoses represent serious psychiatric disorders in which an individual perceptions, thoughts, mood and behavior are significantly altered. Each person who develops a psychosis lives a unique set of symptoms and experiences that may widely vary depending on life circumstances. Although cognitive behavioral psychotherapy (CBT) for psychosis is recommended by main international guidelines, its effectiveness in real-world is still a subject of controversy.

**Objectives:**

The aim of this study was to evaluate, in an Italian outpatient clinical setting, eventual improvements induced by a 6 months intensive CBT specific programme focused on metacognition and emotional regulation and its consequences on psychosocial functioning and subjective well-being in pharmacologically stabilized psychotic patients.

**Methods:**

Eight patients with schizophrenia spectrum disorders (DSM-V), clinically and pharmacologically stabilized, were included in a 6-month program of weekly CBT sessions with focus on metacognition, emotional dysregulation, social functioning and subjective well-being. Patients were assessed with the Metacognitions Questionnaire-30, Difficulties in Emotion Regulation Scale, Heinrichs Quality of Life Scale, The Psychological General Well-Being Index, Brief Psychiatric Rating Scale, Hamilton Depression Rating Scale at baseline and at 3 and 6 months, to verify any improvement on these specific domains and, possibly, on general psychopathology.

**Results:**

In this study CBT showed to be effective on all domains evaluated, most notably for younger patients with a short history of disease (<5 years). Metacognitive capacity was the dimension with most evident improvements, followed by the ability to modulate emotions and the consequent improvement in psychosocial functioning and perceived subjective well-being. During the 6 months follow-up none of the enrolled patients experienced symptoms exacerbation or psychotic relapses.

**Conclusions:**

In conclusion, the 6-month CBT treatment showed to be effective for stabilized psychotic patients, improving metacognitive functions, emotional regulation, psychosocial functioning, and subjective well-being. In addition, insight, adherence and the therapeutic alliance improved. The absence of psychotic relapses is not attributable with certainty to the effect of CBT since, for this purpose, longer duration studies on larger case series and with RCT methods are required. However, it is plausible that the improvement obtained in disease awareness and adherence may be a facilitating factor in relapse reduction.

**Disclosure of Interest:**

None Declared

